# Optical Performance of a Segmented Extended-Depth-of-Focus Intraocular Lens under the Influence of Different Values of Spherical Aberration Generated by Refractive Surgery

**DOI:** 10.3390/jcm12144758

**Published:** 2023-07-18

**Authors:** Luís Salvá, Scott García, Salvador García-Delpech, Anabel Martínez-Espert, Vicente Ferrando

**Affiliations:** 1 Oftalmedic Salvà, 07013 Palma de Mallorca, Spain; lsalva@oftalmedic.com (L.S.); brscottgarcia@gmail.com (S.G.); 2 Clínica Aiken, Fundación Aiken, 46004 Valencia, Spain; 3 Departamento de Óptica y Optometría y Ciencias de la Visión, Universitat de València, 46100 Valencia, Spain; anabel.martinez@uv.es; 4 Centro de Tecnologías Físicas, Universitat Politècnica de València, 46022 Valencia, Spain; viferma1@upv.es

**Keywords:** cataracts, refractive surgery, multifocal IOLs, spherical aberration, adaptive optics visual simulator

## Abstract

Background: Corneal refractive surgery induces high-order aberrations, specifically spherical aberration (SA). These aberrations can have implications when patients later develop cataracts and require the implantation of multifocal intraocular lenses (MIOLs). MIOLs with asymmetric designs pose challenges in predicting outcomes, particularly in these cases. The aim of this study was to evaluate how different values of SA, resulting from various types of refractive surgeries, affect the optical performance of the FEMTIS Comfort intraocular lens. Methods: The through-focus modulation transfer function (TF-MTF) curve and high-contrast images of tumbling E were used as parameters to assess the optical performance of the MIOL. These parameters were measured using an adaptive optics visual simulator. Results: Increasingly negative values of SA make the MIOL more bifocal, moderating its extended-depth-of-focus characteristic. Conversely, higher positive SA values cause the TF-MTF curve to shift towards positive vergences, leading to worsened distance vision in the +1.00 to +2.00 D range, but improved vision in the +0.50 D to +1.00 D range. Conclusions: Assessing SA in patients prior to implanting MIOLs with asymmetric designs is necessary for predicting outcomes and making informed decisions based on the visual requirements of patients.

## 1. Introduction

Corneal refractive surgery is currently one of the most common techniques for correcting visual defects and giving patients independence from spectacles or contact lenses. Different surgical techniques can be employed to perform this procedure: photorefractive keratectomy (PRK), laser-assisted in situ keratomileusis (LASIK), or small-incision lenticule extraction (SMILE). Among these options, LASIK has historically been the most commonly employed due to its swift enhancement of visual outcomes and minimal adverse effects [1]. Nevertheless, studies have indicated that the SMILE technique induces fewer aberrations compared to traditional LASIK [2]. In fact, all these procedures entail reshaping the curvature of the cornea, resulting in alterations in the high-order aberrations of the cornea [3,4]. Although several studies have been conducted to assess the aberrations induced by different refractive surgery techniques [2,5,6,7,8,9,10,11,12], there are no consensus results regarding the changes in individual aberration terms. The differences obtained in different studies may be related to factors such as the levels of preoperative aberrations, the specific surgical technique used, the amount of correction needed, the skill of the surgeon, and obviously the pupil diameter used in the assessment of the results [13]. One of the most prominently impacted aberrations in refractive surgery is the spherical aberration (SA) of the cornea [14]. Across the above-mentioned studies, a consistent finding has emerged: after hyperopia interventions, corneal SA exhibited more negative values [2,5,14], whereas, after myopia correction, SA tended to have more positive values [2,6,7,8,9,10,11,12,14].

Over time, patients who undergo refractive surgery develop presbyopia and cataracts. To address these common conditions and reduce the need for spectacles for clear vision at near and intermediate distances, the treatment is the replacement of the crystalline lens with a multifocal intraocular lens (MIOL). Multifocal corrections operate based on the principle of simultaneous vision, projecting several images (focused and defocused) onto the retina simultaneously. Thus, multifocality comes at the cost of reduced optical quality across all distances [15]. There is a wide range of commercially available MIOLs that come in various designs. These MIOLs can be broadly categorized into two primary groups based on their optical principles: purely refractive lenses and hybrid diffractive–refractive lenses. Additionally, depending on the number of focal points generated by MIOLs, they can be further classified as bifocal, trifocal, or extended-depth-of-focus (EDoF) intraocular lenses (IOLs) [16,17]. While bifocal and trifocal lenses offer multiple focal points, EDoF lenses are defined clinically by the American Academy of Ophthalmology working group [18]. Currently, EDoF IOLs are particularly noteworthy [17]. Previous studies have demonstrated satisfactory outcomes in patients with a history of corneal refractive surgery following the implantation of EDoF IOLs [14,19,20]. Among these lenses, the FEMTIS Comfort IOL (Teleon Surgical B.V., Spankeren, The Netherlands) stands out for its improved stability since it is fixed in the capsulorhexis with specially designed haptics [20]. This is a refractive lens with a rotationally asymmetric near-vision segment on one area of the optic. Thus, refractive rotationally asymmetric multifocal IOLs have emerged as an alternative to concentric ring EDoF IOLs, offering both minimized photic phenomena (haloes and glare) and superior contrast sensitivity [21,22]. Moreover, the stability properties of the FEMTIS Comfort IOL offer the opportunity to achieve customized surgery that would not be possible with a lens featuring a symmetric design, where the position of the IOL inside the capsular bag is not fully predictable.

However, precisely due to this asymmetric design, clinical results with this kind of MIOL are often more difficult to predict than in rotationally symmetric lenses. Furthermore, even after surgery, autorefraction showed a poor correlation with subjective refraction with these radially asymmetric MIOLs [23]. Outcome predictions are even more difficult when SA has to be taken into account and, to our knowledge, there are no studies performed in this field.

Therefore, in this work, the aim is to study the influence of different positive and negative SA values (which arise as a result of refractive surgeries) on the optical performance of the FEMTIS Comfort IOL. To this end, we employed a commercial adaptive optics visual simulator to obtain the through-focus modulation transfer function (TF-MTF) and images of a tumbling E optotype for different object vergences [24]. Since IOLs are typically designed for eyes with a standard corneal geometry and aberrations, it is important to investigate their performance under varying degrees of aberrations in the eye. Visual simulators serve as an excellent tool for assessing visual performance with manipulated aberrations. In fact, unlike computer eye models, which, even if customized to the biometry of the subject, can only predict retinal image quality, visual simulations enable direct measurement of visual performance under manipulated aberrations. Additionally, visual simulators allow for comparing different conditions, including low- and high-order aberrations, within the eye of the same subject.

## 2. Materials and Methods

### 2.1. Multifocal Intraocular Lens Description

**Figure 1:**
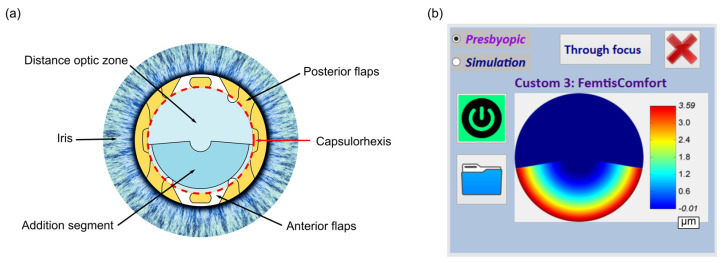
(**a**) Schematic diagram illustrating FEMTIS Comfort intraocular lens fixed in the capsulorhexis. (**b**) Screenshot of the phase (in microns) of the intraocular lens given by the adaptive optics visual simulator.

The FEMTIS Comfort IOL features a biconvex refractive design and an aspheric posterior optic. With a 5.7 mm optical zone diameter, it incorporates a near segment that provides a near addition equivalent of 1.5 D (see Figure 1). Composed of hydrophilic acrylates with a hydrophobic surface, this MIOL has a plate-haptic design with four flaps that fix the optic to the capsulorhexis [25,26]. The low value of the addition allows a partial superposition of two foci resulting in an extended-depth-of-focus IOL [25]. More details are given in Section 2.1.

### 2.2. Experimental Procedure

To evaluate the optical performance of the MIOL, a commercial adaptive optics visual simulator (VAO, Voptica SL, Murcia, Spain) was used [27]. The VAO system incorporates both aberrometry and adaptive optics technology to measure ocular aberrations. Furthermore, this device is capable of simulating various optical profiles or designs for intraocular lenses before refractive surgery, using subjective refraction as the reference baseline. Instead of working with actual patients, we employed an artificial eye with a power of 20 D, which consisted of an achromatic doublet (AC254-050-A-ML, Thorlabs Inc., Newton, NJ, USA) equipped with a CMOS sensor (EO-10012C LE, 8 bits, 3840 × 2748 pixels, 6.41 × 4.59 mm) as previously described in other works. This approach has been utilized to assess different MIOL designs and corneal inlays [24,28,29].

**Figure 2:**
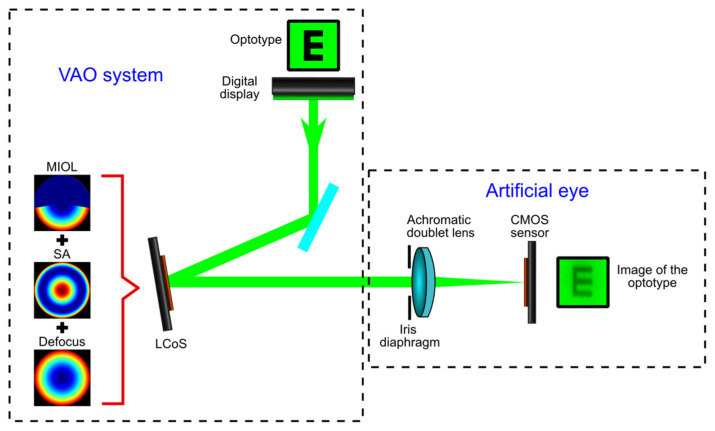
Scheme of an adaptive optics visual simulation and the artificial eye.

The optical setup of the simulator has been described elsewhere [27,30,31,32]. A simplified scheme, depicting the essential elements used in our experiments, is illustrated in Figure 2. A digital display (SVGA micro-OLED) enables the presentation of various visual stimuli (optotypes). The image size is 4.5 × 4.5 mm, sampled at 846 × 846 pixels. A liquid crystal on a silicon spatial light modulator (LCoS) [33] allows for precise placement of the stimuli at the desired distance (i.e., the sphere powers can be changed, to simulate different levels of defocus). In our study, the stimuli were virtually positioned at vergences ranging from −0.50 D to +3.00 D, with intervals of 0.25 D. The LCoS also permits modification of the optical pattern used for registering the stimuli with the artificial eye [34]. In our case, the phase profile of the FEMTIS Comfort IOL [25] and various values of spherical aberration (SA), generated using the simulator, were added to each defocus value during each measurement. The optical performance was evaluated for the nominal working pupil of the instrument: 4.50 mm diameter, under medium photopic luminance (80 cd/m2). The instrument is equipped with software that controls both the digital display and the LCoS, facilitating the easy exploration of the through-focus behaviour of a given MIOL.

Published data on corneal SA values after corneal refractive surgery were considered to determine the different values of SA [2,5,6,7,8,9,10,11,12]. The mean SA value of healthy corneas, SA = +0.28 µm for a 6 mm pupil diameter, was chosen as a reference [35]. This value was specifically scaled for a 4.50 mm pupil diameter [36] to match the pupil size of the VAO system, resulting in SA = +0.09 µm. To evaluate the postoperative behaviour of FEMTIS Comfort IOL in patients undergoing refractive surgery, we considered SA values of 0.0 µm, −0.05 µm, and −0.10 µm to predict the results in hyperopic patients [2,5,14], and SA values of +0.15 µm, +0.20 µm, and +0.25 µm to predict the results in myopic patients [2,6,7,8,9,10,11,12,14].

**Figure 3:**
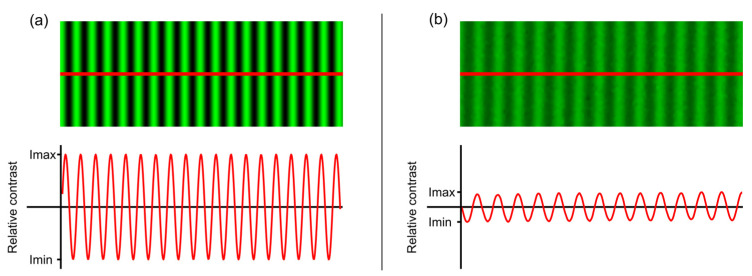
(**a**) Sinusoidal grating test object (upper part). A profile of the corresponding sinusoidal function (red line) is shown below. (**b**) A typical image of the test object provided by the artificial eye and its corresponding sinusoidal profile.

The TF-MTF curve was evaluated by measuring the relative contrast C of the images of a sinusoidal grating test object projected with a green background, using the green channel of the digital display, at different vergences [37], using the Michelson contrast formula: $C=\left(Imax-Imin\right)/\left(Imax-Imin\right)\;$(see Figure 3). The grating had a spatial frequency of 19 cycles/degree, equivalent to, approximately, a 0.2 logMAR visual acuity (VA) optotype. This specific spatial frequency was chosen because it is the reference VA value used by the American Academy of Ophthalmology for classifying an IOL as an EDoF lens [18].

In addition to the TF-MTF evaluation, through-focus images of high-contrast tumbling E optotypes corresponding to VA values of 0.4 logMAR, 0.2 logMAR, and 0.0 logMAR were also obtained.

## 3. Results

**Figure 4:**
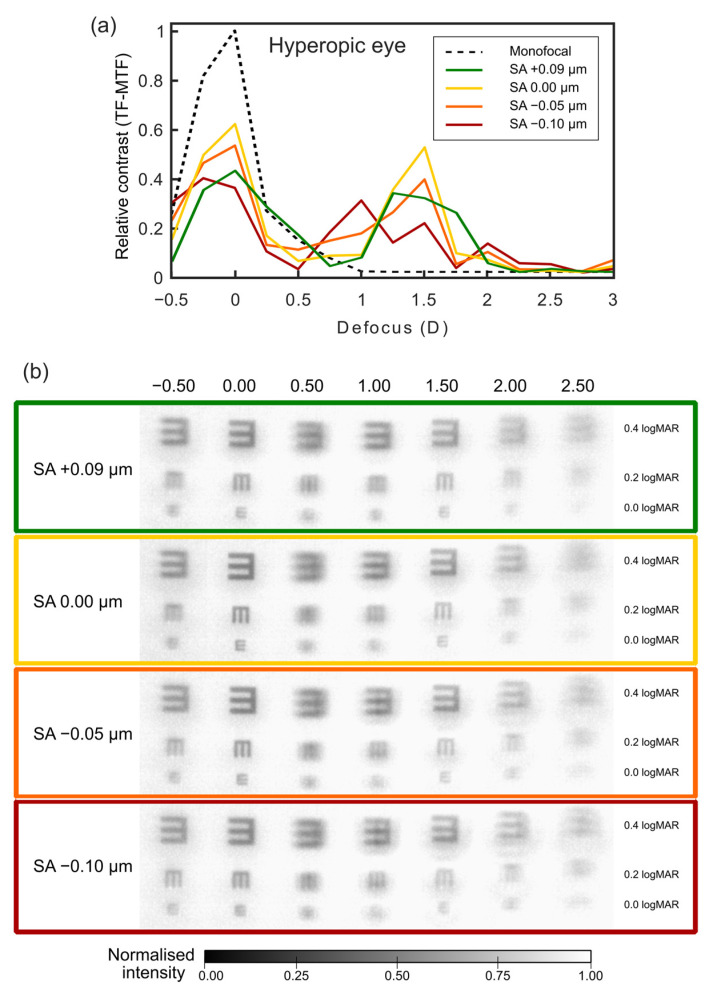
(**a**) Relative contrast (through-focus modulation transfer function (TF-MTF)) as a prediction of the spherical aberration (SA) effect on hyperopic eyes after surgery. The experimental values were normalized to those obtained with a monofocal lens, which is represented with a black dashed line. (**b**) Through-focus images of a tumbling E optotype provided by the FEMTIS Comfort intraocular lens with induced different SA values related to hyperopia refractive surgery for a pupil size of 4.50 mm.

**Figure 5:**
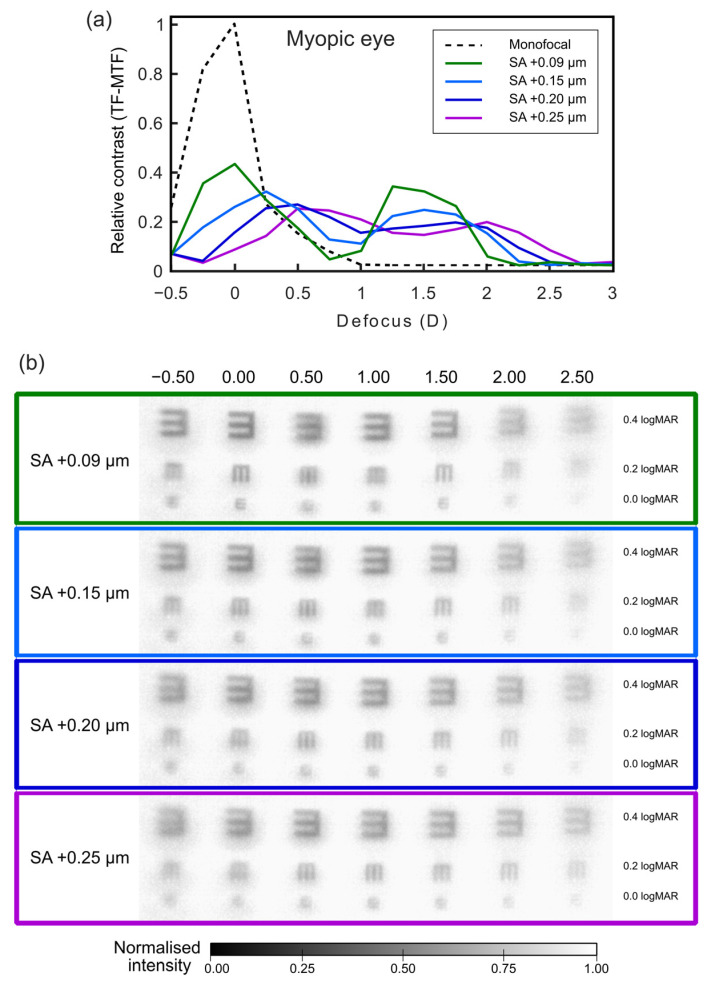
(**a**) Relative contrast (TF-MTF) as a prediction of the SA effect on myopic eyes after surgery. The experimental values were normalized to those obtained with a monofocal lens which is represented with a black dashed line. (**b**) Through-focus images of a tumbling E optotype provided by the FEMTIS Comfort intraocular lens with induced different SA values related to hyperopia refractive surgery for a pupil size of 4.50 mm.

Figure 4 and Figure 5 show the TF-MTF values and the corresponding through-focus images of high-contrast tumbling E optotypes corresponding to different VA values. In Figure 4a and Figure 5a, the TF-MTF for the eye corrected with a monofocal IOL and zero SA is also shown for comparison. The relative contrast was obtained by the ratio between the measured values of contrast and the contrast obtained with the eye corrected with a monofocal IOL and zero SA, which was set to 1.

As mentioned before, the value SA = + 0.09 µm was taken as a reference in both figures. It can be seen that with this SA value, the TF-MTF (green line) of the FEMTIS Comfort IOL presents a clear bifocal profile with one focus for distance vision (defocus = 0.0 D) and another one with a slightly lower value (around 20%) for intermediate vision (defocus = +1.25 D).

The corresponding trough-focus images shown in Figure 4b and Figure 5b inside the green frame also reflect this behaviour: The best images can be observed at the two main foci. In both foci, all three letters of the optotype can be resolved, resulting in a VA of 0.0 logMAR. For vergences between the main foci, the E corresponding to VA 0.2 logMAR is recognisable.

### 3.1. Prediction of the SA Effect on Hyperopic Eyes after Surgery

Since the cornea becomes more prolate after corneal refractive surgery to correct hyperopia, the SA tends to acquire more negative values than before the surgery. Thus, in order to predict the results in hyperopic patients, the reference TF-MTF curve and the corresponding images for SA values of 0.0 µm, −0.05 µm, and−0.10 µm were compared in Figure 4.

It can be seen that for SA values of 0.0 µm, the TF-MTF values increase at both foci. However, the lens becomes more prominently bifocal, obtaining worse values in the range from +0.25 D to +0.75 D. For the TF-MTF corresponding to SA = −0.05 µm, the same occurs as in the previous case in the two main foci, although a slight decrease is observed with respect to SA = 0.0 µm. In this case, the reference value is still better in the range from +0.25 D to +0.60 D, but decreases more steeply at +1.75 D. Finally, for SA = −0.10 µm, a focal shift of -0.25D in the far focus and a decrease in the TF-MTF values within the range from +1.25D to +1.75D are observed

The effects of different values of SA on the corresponding VA can be seen in Figure 4b. In this figure, the colour of the frames corresponds to that of the curves in Figure 4a. With an SA value of 0.0 µm, visibility at the foci increases, but the EDoF condition is lost, as the 0.2 logMAR letters are not resolved for +0.50 D defocus. For SA values of −0.05 µm and −0.10 µm, both the image resolution at the foci and the bifocal profile decrease. These results align with the trends observed in the TF-MTFs.

As a general rule, it can be deduced that more negative values of SA induced by refractive surgery in hyperopic eyes tend to accentuate the bifocality of the MIOL by improving the foci in relation to the reference value, but at the expense of the extended focus concept.

### 3.2. Prediction of the SA Effect on Myopic Eyes after Surgery

After corneal refractive surgery to correct myopia, the cornea becomes more oblate, and SA tends to adopt more positive values. Thus, in order to predict the results in myopic patients, the reference TF-MTF curve and the corresponding images for SA values of +0.15 µm, +0.20 µm, and +0.25 µm were compared in Figure 5. As the level of induced positive SA increases (Figure 5a), the foci shift towards real vergences, and although the bifocal profile is maintained, the TF-MTF value decreases.

With an SA of +0.15 µm, the curve shifts towards positive vergences. In this case, there is a focus at +0.25 D and another at +1.50 D, both of lower magnitude than the reference value. In the ranges from −0.50 D to +0.25 D and from +1.00 D to +1.75 D, the relative contrast is lower, but the opposite happens in the range from +0.25 D to +1.00 D. For SA = +0.20 µm, the curve shifts further towards positive vergence, but bifocality is maintained. In this case, the best focus is at +0.5 D, and the focus for intermediate vision remains at +1.75 D. Note that from +0.30 D to about +1.00 D, the TF-MTF values are even higher than for the reference curve, and the same happens for defocus greater than +1.90 D. The tendency to shift the curve towards nearer vergences is accentuated for SA = + 0.25 µm values. The highest contrast foci are at +0.50 D and +2.00 D. There is better visibility than in the reference curve in the ranges from +0.40 D to +1.10 D and +1.90 D to +2.75 D.

In Figure 5b, the through-focus images reveal a notable shift in the foci towards real vergences, accompanied by a reduction in the bifocal profile. Additionally, as the SA value increases, there is a decrease in the resolution of the letters at the mean foci, while an increase is observed at intermediate vergences. These findings are consistent with the trends observed in the TF-MTFs.

In summary, the general trend for myopic eyes undergoing refractive surgery is that as the induced SA value becomes higher, the defocus curve shifts to positive vergence, worsening vision at a distance and in the +1.00 D to +2.00 D range, which corresponds to the intermediate/near vision range. By contrast, visibility in the range between +0.50 D and +1.00 D is better.

## 4. Discussion

It is well known that refractive surgery induces different types of higher-order corneal aberrations. In particular, clinical studies have shown that SA values become more positive in myopic eyes and more negative in hyperopic eyes after corneal refractive surgery [5,6,7]. However, it is important to bear in mind that measurements of high-order aberrations are related to pupil size and, consequently, the impact of high-order aberrations on visual performance is more important in mesopic conditions than in photopic ones. Considering that over the years, all these patients will develop presbyopia, with a high rate of cataract development, it is necessary to be able to predict the visual performance of operated eyes when the crystalline lens is replaced by a MIOL. Predicting visual performance becomes especially challenging in refractive MIOLs with asymmetric designs, such as the FEMTIS Comfort IOL. Therefore, in this work, we assessed the optical performance of eyes with different values of SA by means of experimental TF-MTF curves, obtained with a customized visual simulator based on adaptive optics. This system is widely accepted as a useful tool for assessing the visual performance of IOLs and aiding in clinical decisions [38]. Through the experimental TF-MTF and image simulation, we have demonstrated that the FEMTIS Comfort IOL does not behave equally in eyes with positive and negative induced values of SA, and this discrepancy would be reflected in the results of cataract surgery performed on eyes with prior myopic and hyperopic refractive surgery. Our results indicate that this lens would perform better if implanted in patients with corneas that have undergone hyperopic refractive surgery (Figure 4) compared to those who have had myopic refractive surgery (Figure 5), due to the fact that the evaluated IOL in this study does not induce SA. Moreover, patients undergoing myopic refractive surgery (Figure 5) may also experience worse vision at far distances, as inducing SA causes a shift of the foci towards nearer vergences. In other words, since in myopic eyes, refractive surgery increases positive SA, the use of IOLs with negative SA is recommended to reduce SA values, and, on the other hand, since after refractive surgery for hyperopia, negative SA values are induced in the cornea, the use of IOLs with zero SA is recommended [19].

Some limitations of this study mean that the results obtained, while qualitatively relevant, cannot be generally extrapolated to clinical practice. The main limitations include the following: (i) The use of monochromatic light. We have chosen this option to minimize the influence of chromatic aberrations on the measurements and their interaction with SA. Using polychromatic illumination, the effect of longitudinal chromatic aberration extends the depth of focus, masking the net effect of the SA [39,40]. (ii) The use of an artificial eye for capturing the TF-MTFs with the visual simulator. In a typical clinical study, different groups of subjects would be categorized based on factors such as the magnitude of their corneal aberrations. However, the results of such studies may be affected by variations in neural response among different individuals, leading to potential confounding factors. To mitigate this, we performed our experiments with a customized visual simulator, allowing for virtual intra-subject testing with different amounts of SA. This approach helps minimize the impact of inter-subject variability in neural response, enabling more accurate comparisons and assessments [24].

On the other hand, although the study was conducted with the lens oriented as shown in Figure 1, altering the lens orientation would not have a significant impact on our results due to the rotational symmetry of SA. In fact, it has been shown that asymmetrical designs with different orientations did not significantly change visual acuity [41]. However, the perceptual orientation preferences, tested using a simultaneous vision simulator, varied across subjects and vergences. Therefore, the optimum orientation was driven by the interactions of the design with the optical aberrations of the eye. So, the preference for a certain orientation would be related to the high-order aberration of the patient.

Nevertheless, it is important to note that other non-symmetric higher-order aberrations, such as coma, may have more pronounced effects. As we mentioned in the introduction, the effects of higher-order aberrations are eye-dependent and unpredictable in general. Therefore, in this study, we have focused on the effects of SA, and we did not assess the impact of coma and astigmatism on the performance of the FEMTIS Comfort IOL, nor did we consider the neuroadaptive response. However, it is important to note that an experimental study demonstrated the potential for IOLs with negative SA to effectively compensate for horizontal coma [42]. This compensation is likely attributable to the oblique incidence of rays, which induces horizontal coma in the cornea but also generates coma in the lens with an opposite sign to that of the cornea. Notwithstanding, we want to point out that other high-order aberrations can be investigated using the same methodology employed in this work. In particular, due to the high stability of the FEMTIS Comfort IOL, highly predictable results could be obtained, and testing the lens with different orientations could be explored to customize the surgery based on individual needs. In this sense, we would like to emphasize the importance of evaluating aberrations in patients prior to the implantation of MIOLs with asymmetric designs, especially in those who have undergone refractive surgery. At this point, it is important to note that, in the case of MIOLs with symmetric designs, especially those with aspheric profiles, clinical outcomes could be predicted using the same approach as employed in this work.

In conclusion, despite the aforementioned limitations, the results presented in this paper offer valuable objective insights for surgeons, because these assessments play a critical role in making informed decisions that align with the visual needs of patients. Furthermore, these findings suggest that the use of adaptive optics simulators in these patients can be highly beneficial in preventing unfavourable outcomes post-surgery. Informing patients about the available choices and discussing the potential effects of their chosen MIOL on their ocular health and lifestyle is crucial to ensure the success of an intervention and patient satisfaction.

## Data Availability

The data that support the findings of this study are available from the corresponding author, S.G.-D., upon reasonable request.
